# Supplementary material for: Engineering crystalline quasi-two-dimensional polyaniline thin film with enhanced electrical and chemiresistive sensing performances
Source: Nat Commun. 2019 Sep 23;10:4225. doi: 10.1038/s41467-019-11921-3 (PMC6757049; doi:10.1038/s41467-019-11921-3)
Supplement: Supplementary file 1 — Supplementary Information [file 41467_2019_11921_MOESM1_ESM.pdf]

# Supplementary Information

## Engineering Crystalline Quasi-Two-Dimensional Polyaniline Thin Film with Enhanced Electrical and Chemiresistive Sensing Performances

Zhang et al.

## Supplementary Figures

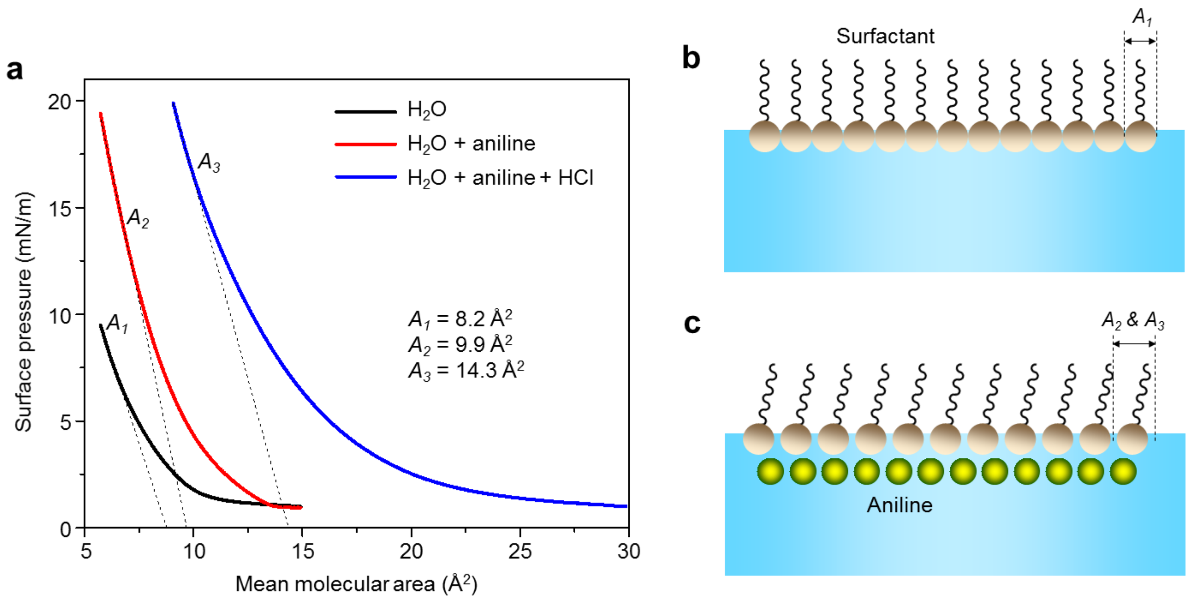


**Supplementary Figure 1 | Surface pressure-mean molecular area (*π*-*A*) isotherms of sodium oleyl sulfate.** (**a**) *π*-*A* isotherms of the surfactant monolayer on different subphase, e.g., pure water, aniline-water solution, and aniline-HCl-water-solution, respectively. (**b**) Schematic demonstration of the monolayer surfactant on pure water surface. (**c**) Schematic demonstration of the surfactant monolayer on aniline water surface, in which the mean molecular area *A* was increased due to the interaction between the head group (sulfate) of surfactant and aniline.

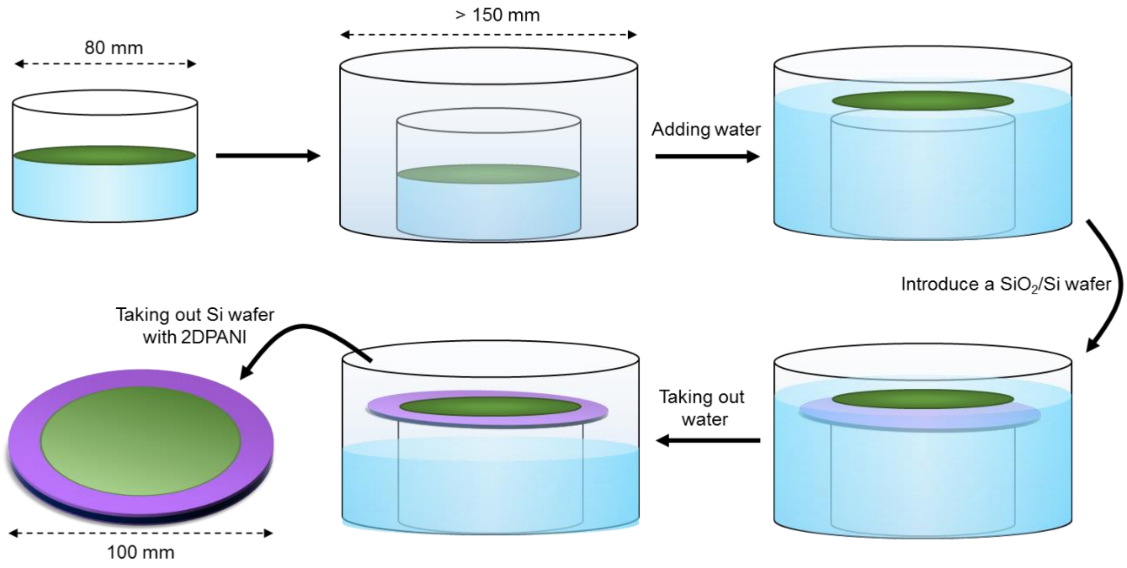


**Supplementary Figure 2 | Schematic demonstration the transfer of q2D PANI onto 4-inch $SiO_2$ wafer.** After polymerization, a uniform film of q2D PANI was formed on the water surface of a glass well (diameter Ø = 8 cm). Afterwards, the glass well was placed in larger one with diameter Ø = 15 cm, followed by adding water till that the q2D PANI film floated onto the top water surface. Afterwards, a 300 nm $SiO_2$/Si wafer (4-inch, Microchemicals GmbH) was placed under the film, and the water was removed slowly using a plastic dropper. As such, the q2D PANI dropped slowly to the wafer surface. The resultant q2D PANI on $SiO_2$/Si was rinsed by chloroform and ethanol, and then dried in vacuum oven for 24 h.

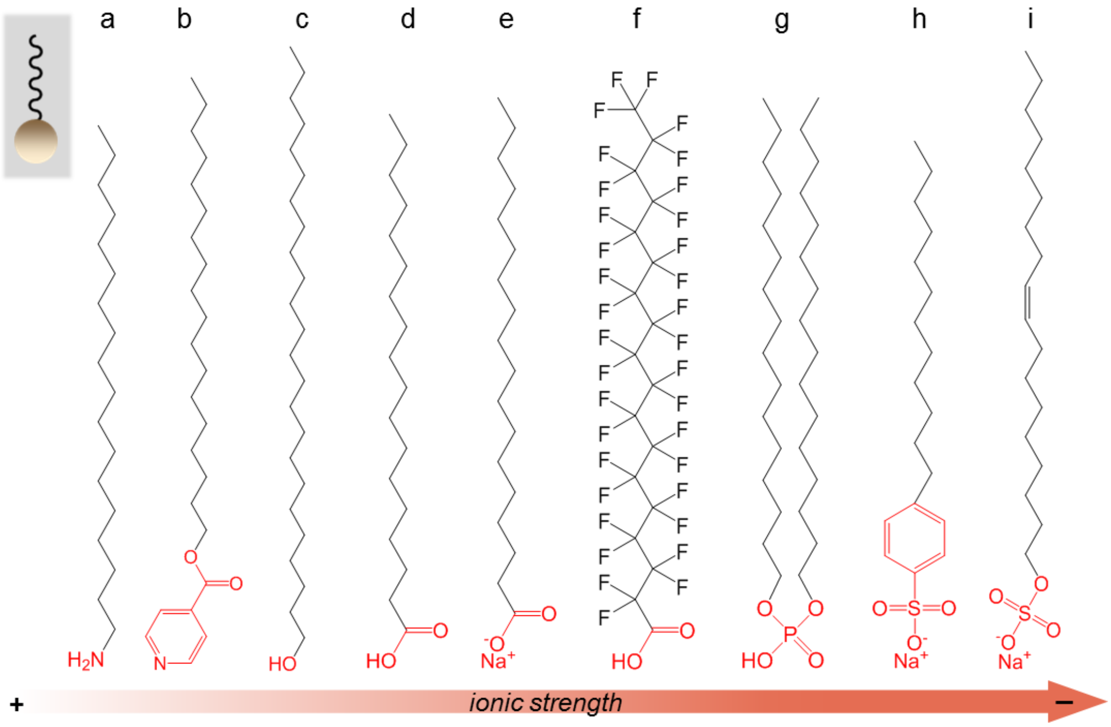


**Supplementary Figure 3 | Surfactants used for the synthesis of q2D PANI.** a, octadecylamine; b, hydrogen ionophore IV; c, lignoceryl alcohol; d, stearic acid; e, sodium stearate; f, perfluorooctadecanoic acid; g, dihexadecyl phosphate; h, sodium dodecylbenzenesulfonate; i, sodium oleyl sulfate.

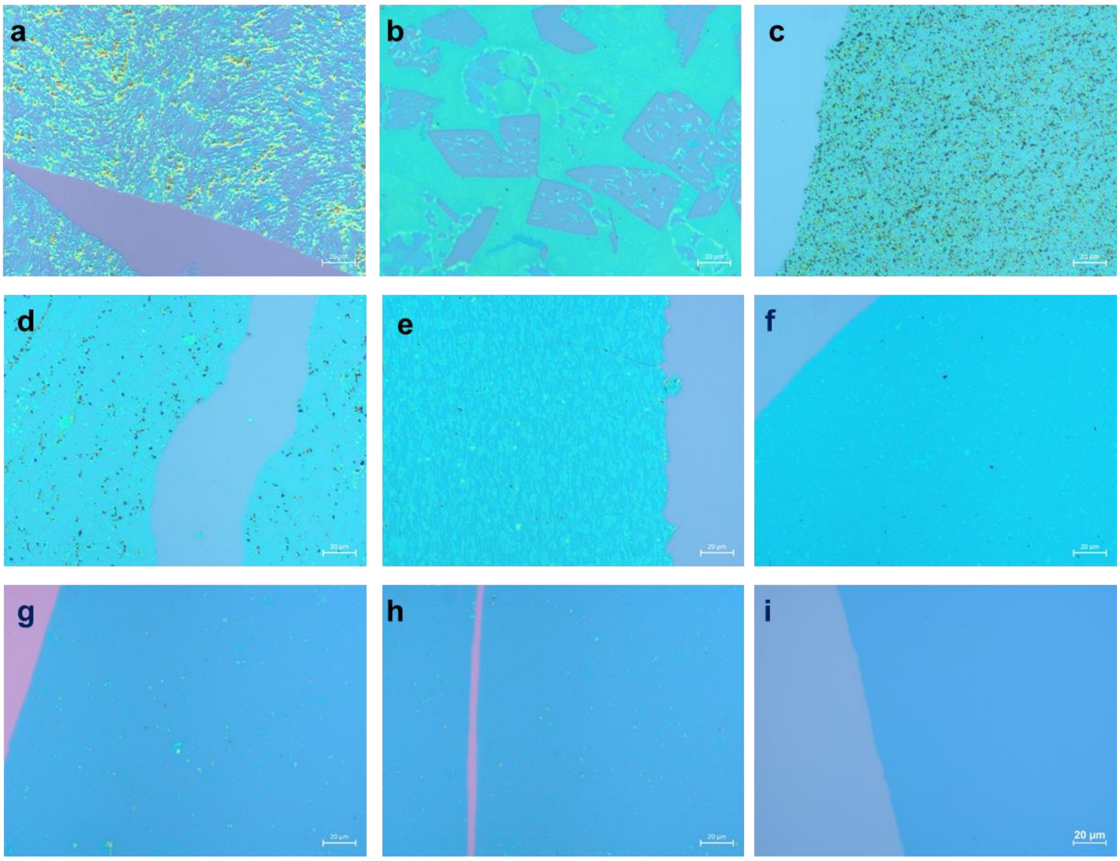


**Supplementary Figure 4 | Optical microscopy images of PANI films prepared with different surfactants**. (**a**) octadecylamine; (**b**) hydrogen ionophore IV; (**c**) lignoceryl alcohol; (**d**) stearic acid; (**e**) sodium stearate; (**f**) perfluorooctadecanoic acid; (**g**) dihexadecyl phosphate; (**h**) sodium dodecylbenzenesulfonate; (**i**) sodium oleyl sulfate. The structures of the surfactants are shown in Supplementary Fig. 3. All the films were prepared in 48 h reaction and 0.02 M HCl. Scale bars, 20 µm.

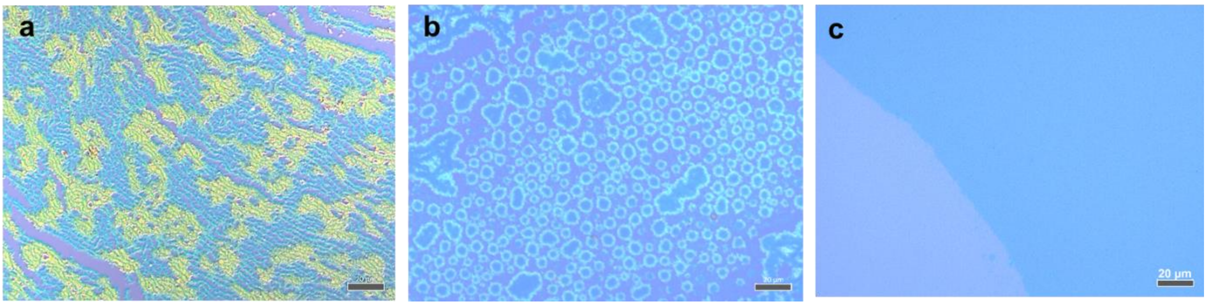


**Supplementary Figure 5 | Optical microscopic images of PANI films**. The films were prepared at (**a**) the pure air-water interface, (**b**) air-water interface with 2 µL (1 mg $mL^{-1}$) SOS, (**c**) air-water interface with 7 µL (1 mg $mL^{-1}$) SOS. Scale bars: 20 µm. According to initial isotherm test, the minimum amount of SOS to form a continuous SOS monolayer on water surface (with diameter Ø = 6 cm) is ~7 µL. Therefore, when the amount of SOS decreased to 2 µL (1 mg/mL) at the air-water interface, the resultant PANI film is rough and discontinuous. In contrast, the resultant PANI film became highly uniform and continuous, when the amount of SOS increased to 7 µL or even more.

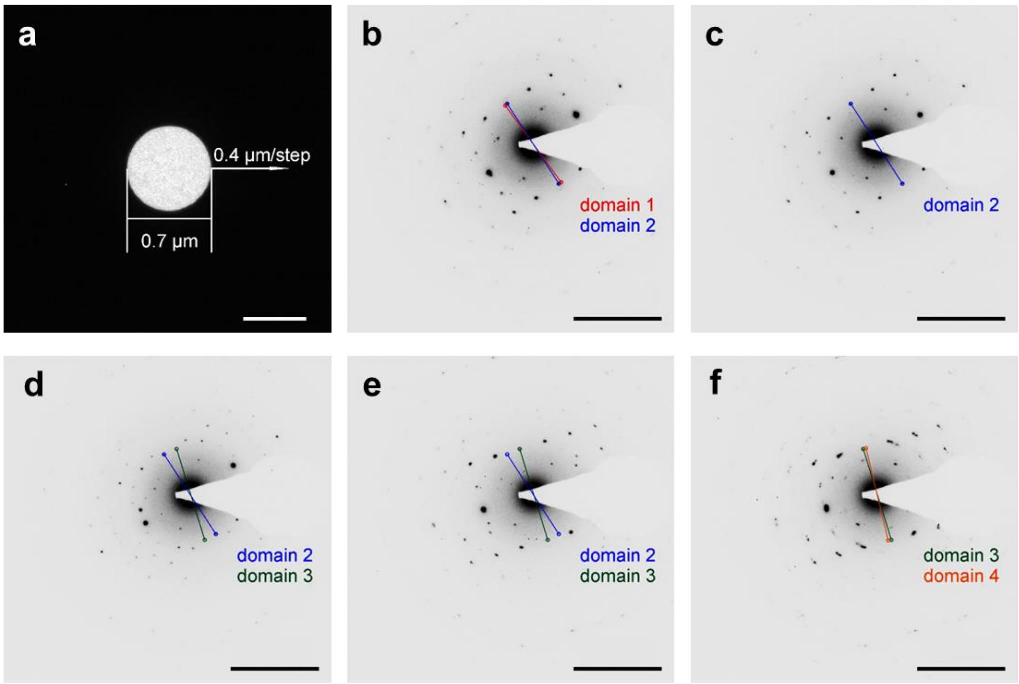


**Supplementary Figure 6 | Evaluation of single-crystalline domain size of q2D PANI using SAED.** (**a**) TEM image of the selected area for the acquisition of SAED patterns. The diameter of the selected circular area is 0.7 μm. The arrow indicates the lateral movement of specimen stage in 0.4 μm step. (**b - f)** Consecutively acquired SAED patterns coupled with specimen stage movement. The orientation of each domain is represented by the colored solid lines. Scale bars: (**a**) 0.5 μm; (**b-f**) 5 $nm^{-1}$.

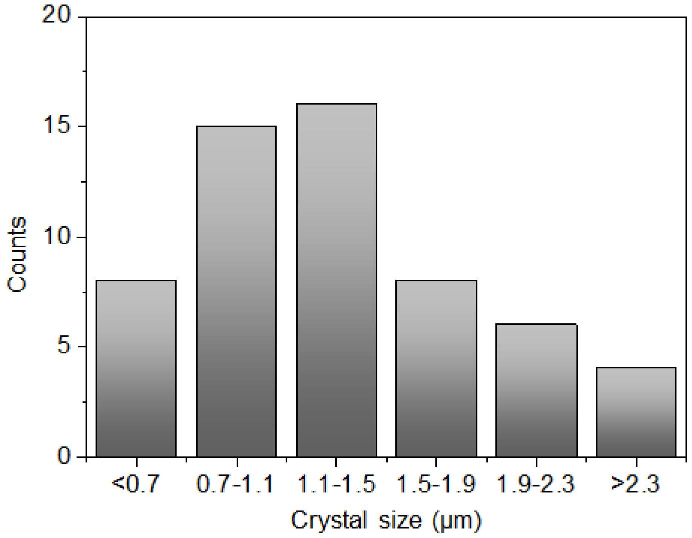


**Supplementary Figure 7 | Histogram of domain size of the q2D PANI derived from 50 domains by SAED.**

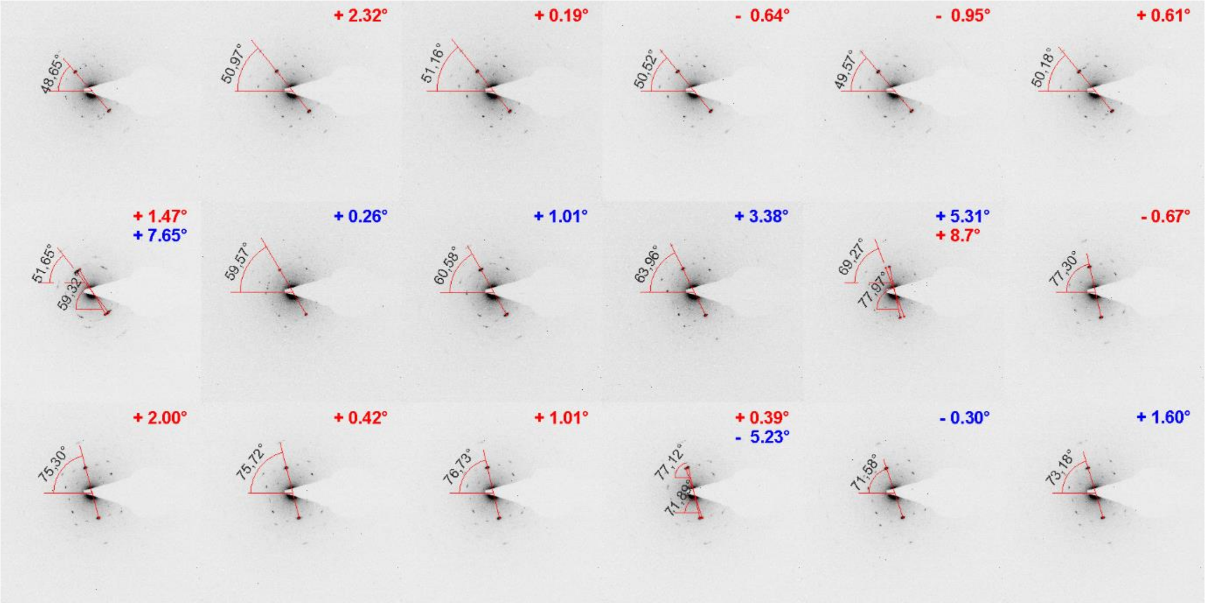


**Supplementary Figure 8 | Rotations of the q2D PANI single crystal domains continuously revealed by SAED.** TEM camera size: 0.7 μm. Each image was taken by continuous and lateral movement of specimen stage in 0.4 μm step. The number on the top right of each image is the rotation degree in comparison to the previous image. The color (red and blue) change implies the appearance of a new crystal, since two sets of SAED single crystal emerge.

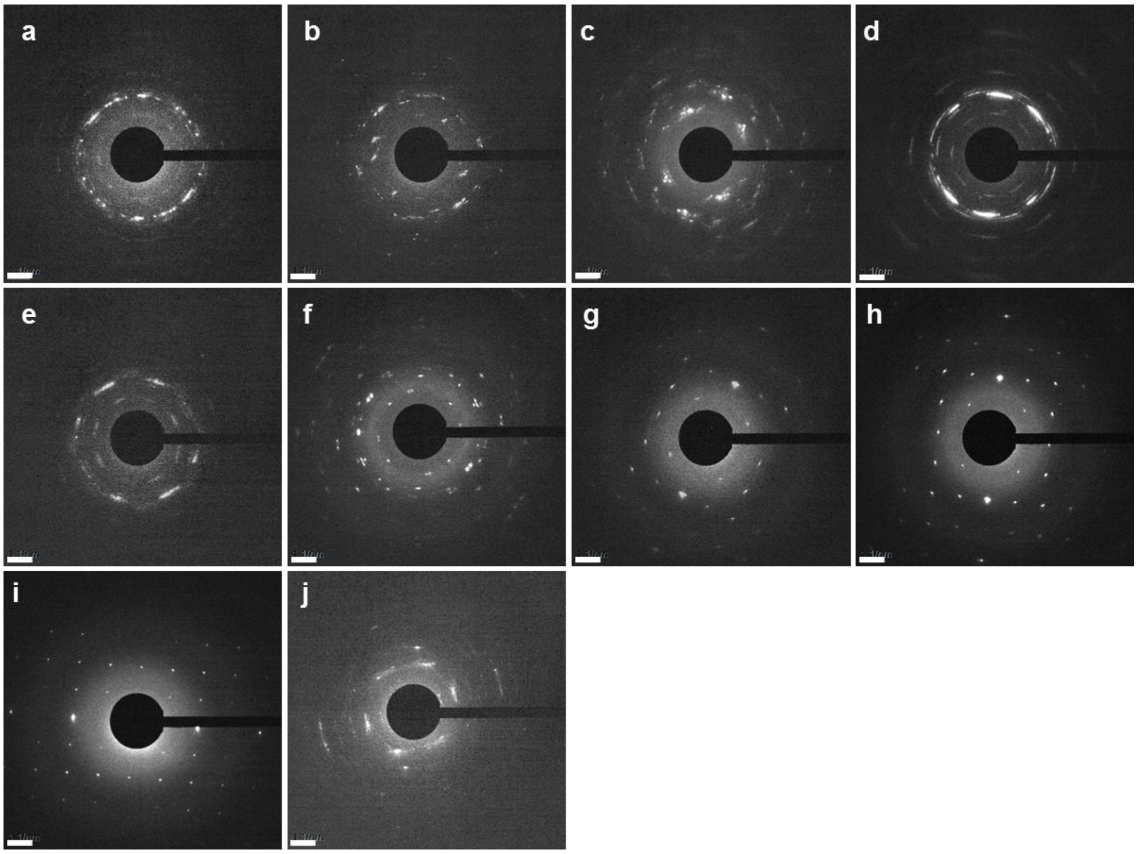


**Supplementary Figure 9 | SAED patterns of PANI films prepared by using various surfactants.** (**a**) octadecylamine; (**b**) hydrogen ionophore IV; (**c**) lignoceryl alcohol; (**d**) stearic acid; (**e**) sodium stearate; (**f**) perfluorooctadecanoic acid; (**g**) dihexadecyl phosphate; (**h**) sodium dodecylbenzenesulfonate; (**i**) sodium oleyl sulfate; (**j**) no surfactant. The structures of the surfactants are shown in Supplementary Fig. 3. Scale bars, 1 $nm^{-1}$.

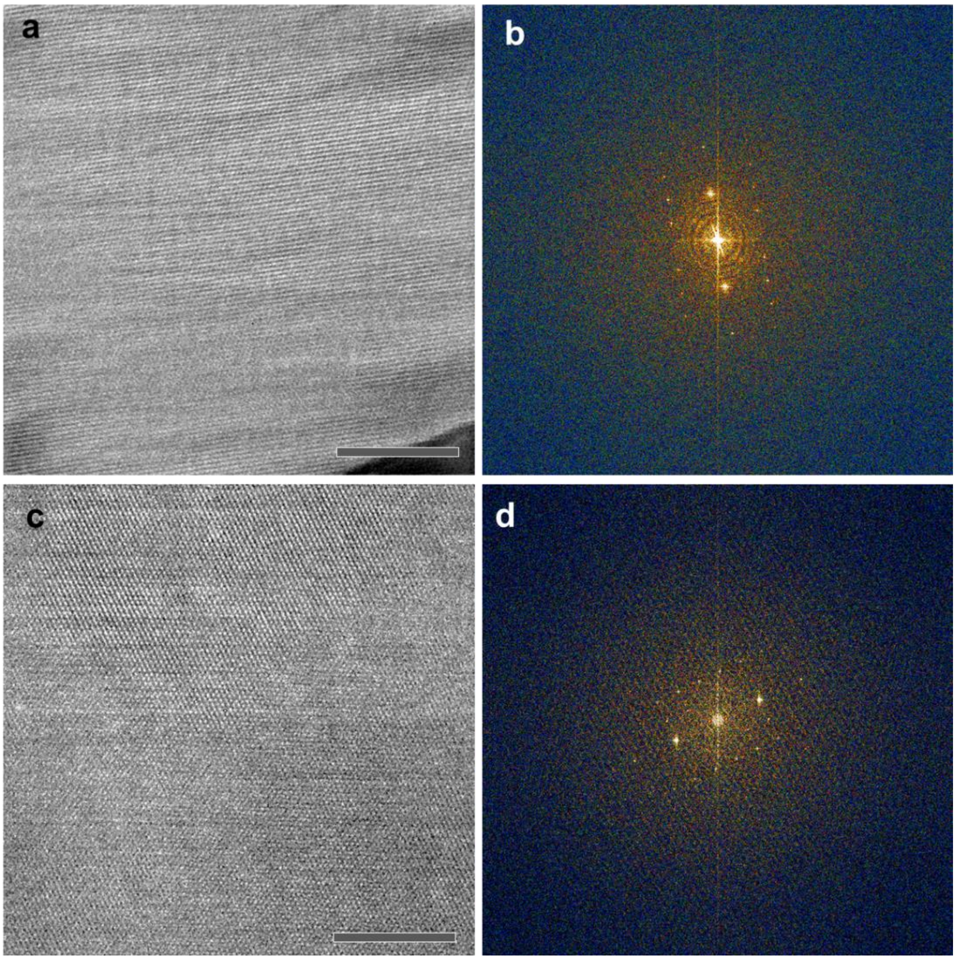


**Supplementary Figure 10 | AC-HRTEM images of single crystalline q2D PANI at different positions**. (**a**) and (**b**) AC-HRTEM images. (**b**) and (**d**) Corresponding FFT images. Scale bars: (**a**) and (**c**), 10 nm.

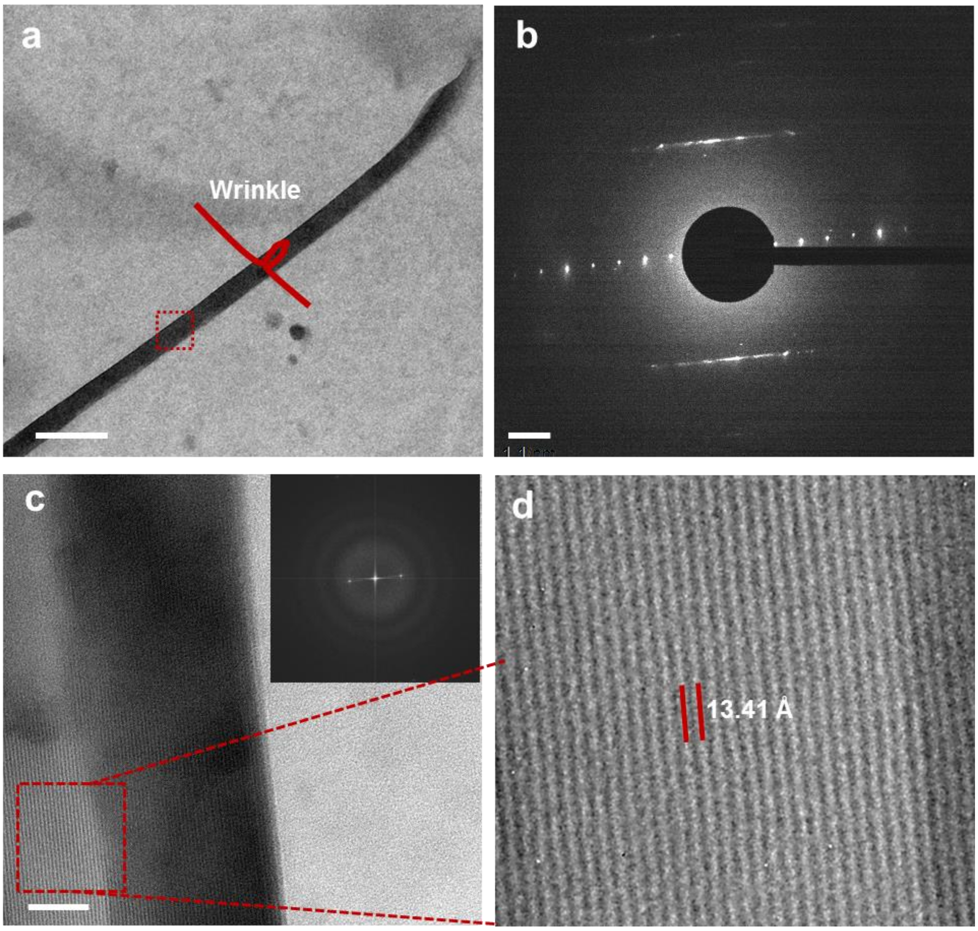


**Supplementary Figure 11 | TEM Characterization.** (**a**) TEM image of a wrinkle position selected on q2D PANI. (**b**) SAED pattern and (**c-d**) HRTEM image of the wrinkle, which is perpendicular to the [001] axis and implies an interplanar spacing of 13.41 Å.

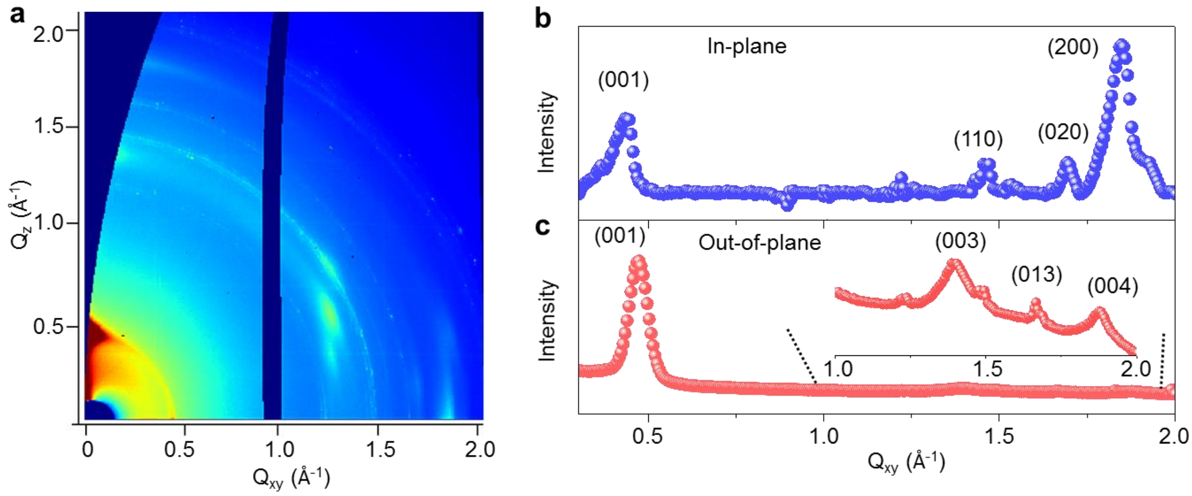


**Supplementary Figure 12 | GIWAXS characterization of q2D PANI film**. (**a**) GIWAXS patterns of ~30 nm q2D PANI on $SiO_2$/Si wafer**.** (**b**) In-plane and (**c**) out-of-plane projections from (**a**). The position of the 001 peak depends on the orientation of crystallites with respect to the substrate plane. The peak position is slightly shifted in-plane and out-of-plane, that is because of the high detector tilt angle which leads to some degree of freedom while calibration.

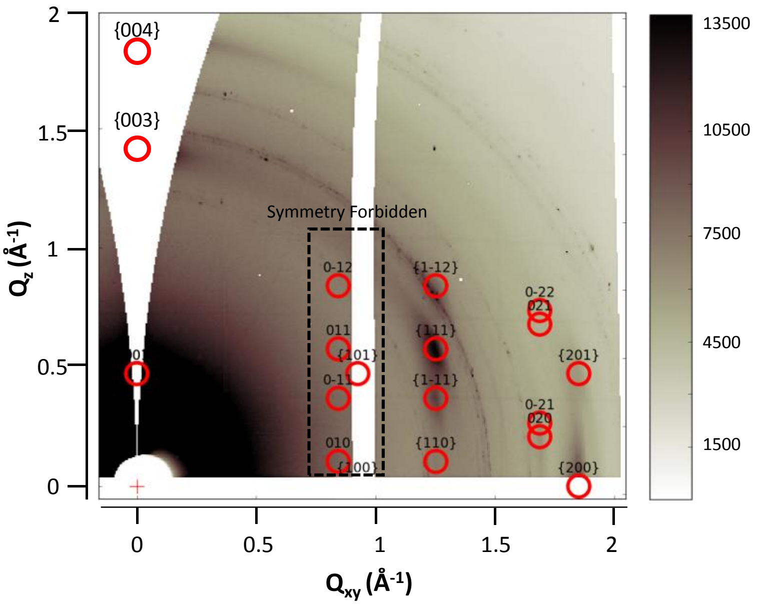


**Supplementary Figure 13 | GIWAXS patterns of q2D PANI and unit cell simulation.** The *p2gg* plane group symmetry is verified since the odd order (*h00*) and (*0k0*) peaks are absent in GIWAXS.

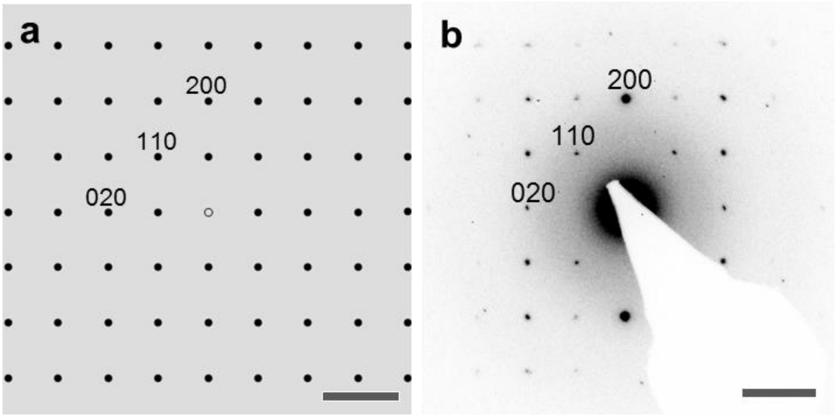


**Supplementary Figure 14 | SAED diffraction patterns of (a) simulated and (b) experimental q2D PANI**. Scale bars: 2 $nm^{-1}$.

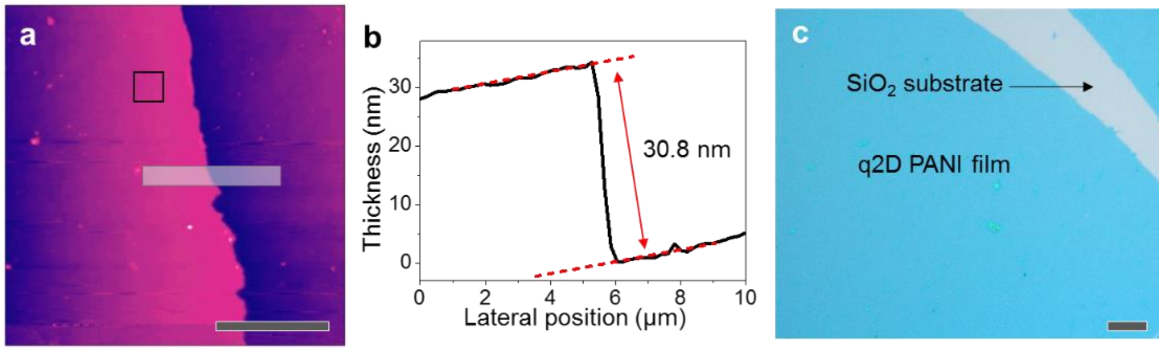


**Supplementary Figure 15 | Characterization of q2D PANI film of 30.8 nm thick**. (**a**) AFM topographic image of q2D PANI film. The area of black box was selected to calculate root-mean-square (RMS) roughness (RMS = ~0.9 nm). (**b**) Corresponding height profile of the grey line in (a). (**c**) Optical microscopic image of the q2D PANI film. Scale bars: (**a**) 10 µm; (**c**) 20 µm. The film was prepared with seven days reaction at air-water interface with SOS surfactant monolayer.

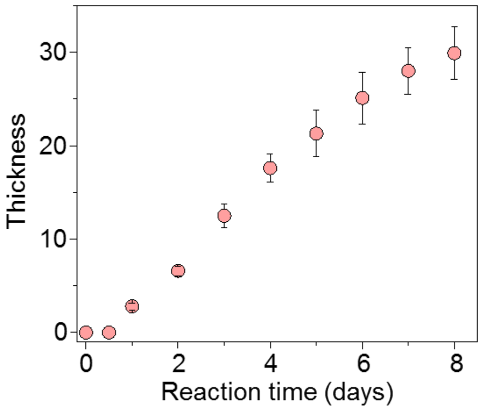


**Supplementary Figure 16 | Growth kinetic of q2D PANI at 1 M HCl under sodium oleyl sulfate monolayer.** The thickness was measured with variable angle spectroscopic ellipsometry. Error bars indicate the variations in thickness of each q2D PANI sample at five different positions.

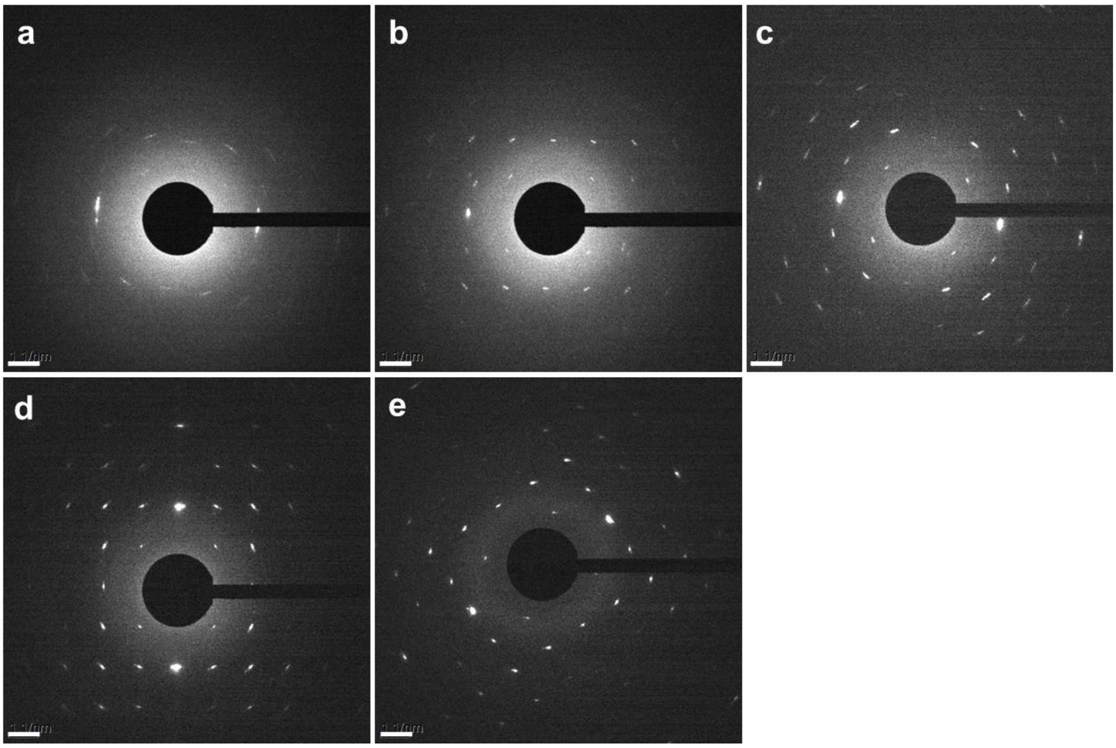


**Supplementary Figure 17 | SAED patterns of q2D PANI films of different thickness.** (**a**) 2.6 nm (12 h); (**b**) 5.8 nm (24 h); (**c**) 10.5 nm (48 h); (**d**) 19.6 nm (4 days); (**e**) 30.8 nm (7 days). Scale bars, 1 $nm^{-1}$.

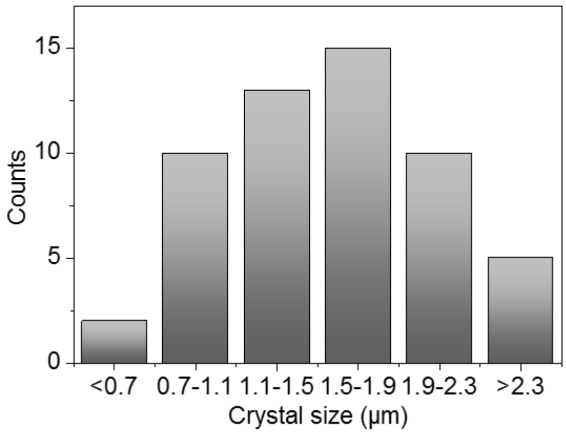


**Supplementary Figure 18 | Histogram of domain size of the q2D PANI of 30.8 nm thick.** The result was derived from 55 domains by SAED.

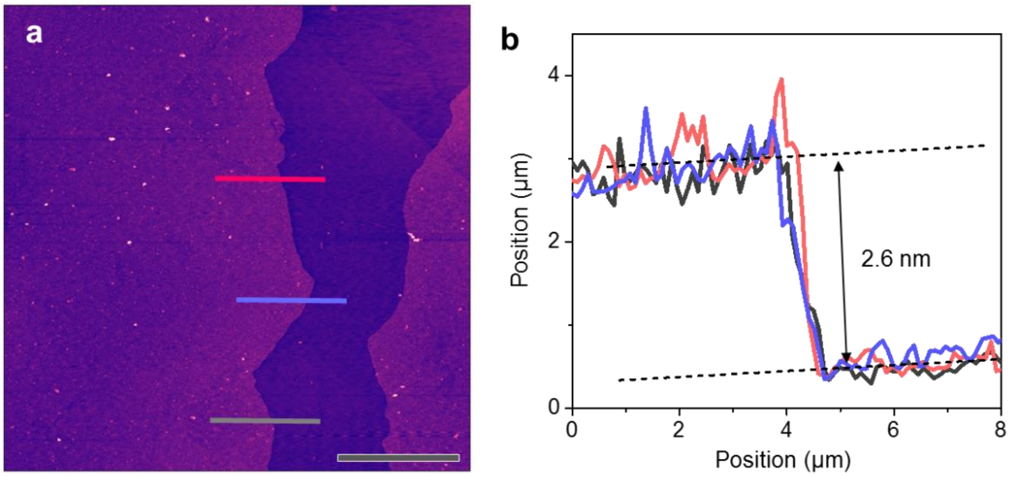


**Supplementary Figure 19 | AFM characterization**. (**a**) AFM topographic image and (**b**) corresponding height profiles of the q2D PANI synthesized at the air-water interface using sodium oleyl sulfate monolayer. Reaction time: 12 h. Scale bar, 10 μm.

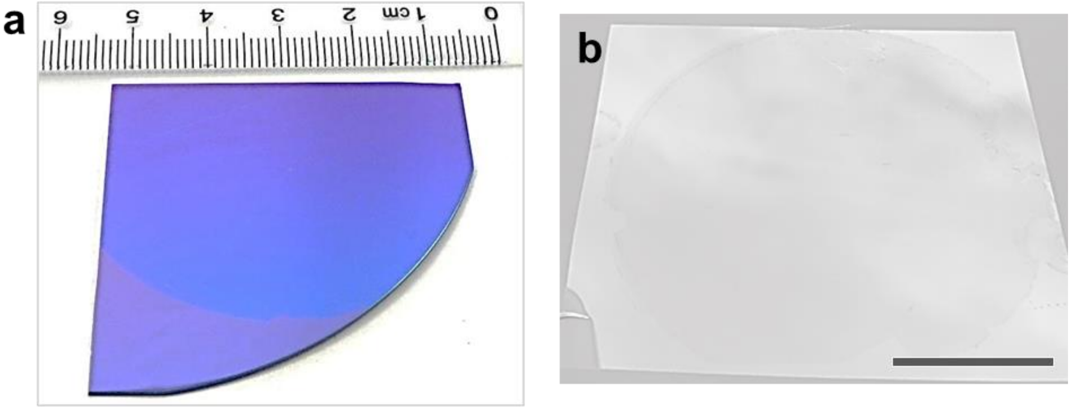


**Supplementary Figure 20 | Photographs of the q2D PANI with 2.6 nm thickness**. Large area continuous q2D PANI film can be transferred onto (**a**) $SiO_2$/Si and (**b**) polyethylene terephthalate (PET) substrates, respectively. Scale bar: (**b**) 2 cm.

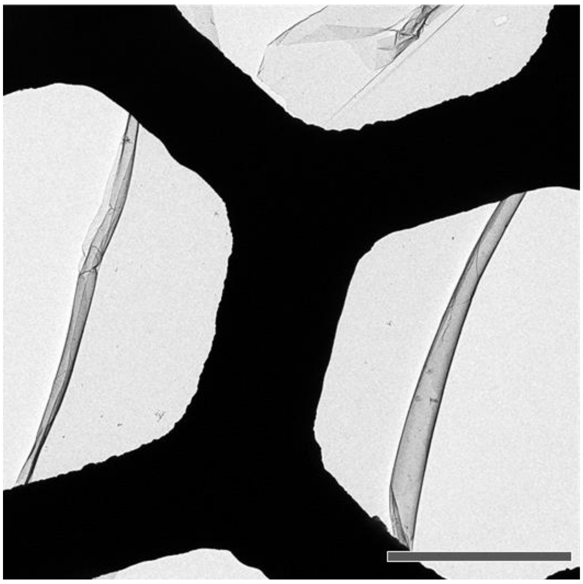

**Supplementary Figure 21 | Free-standing q2D PANI of 2.6 nm thick on 400 mesh TEM grid.** The ultrathin film was ruptured and crimped during measurement due to electron irradiation during TEM imaging. Scale bar, 10 μm.

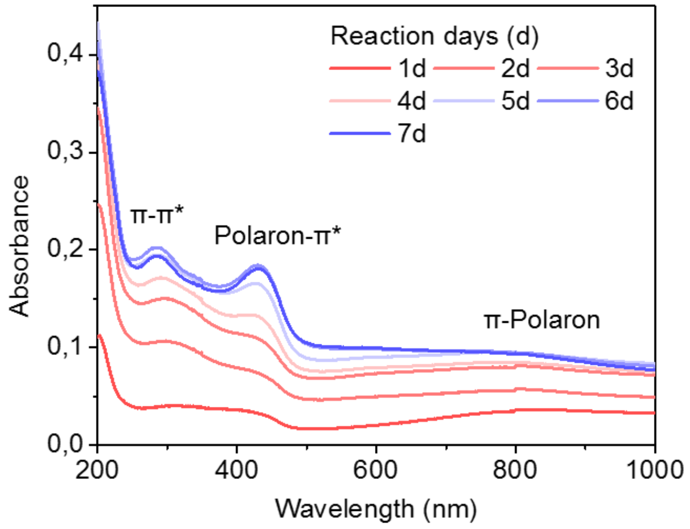


**Supplementary Figure 22 | UV-Vis-NIR absorption spectra.** The q2D PANI films were prepared at various reaction time using sodium oleyl sulfate monolayer.

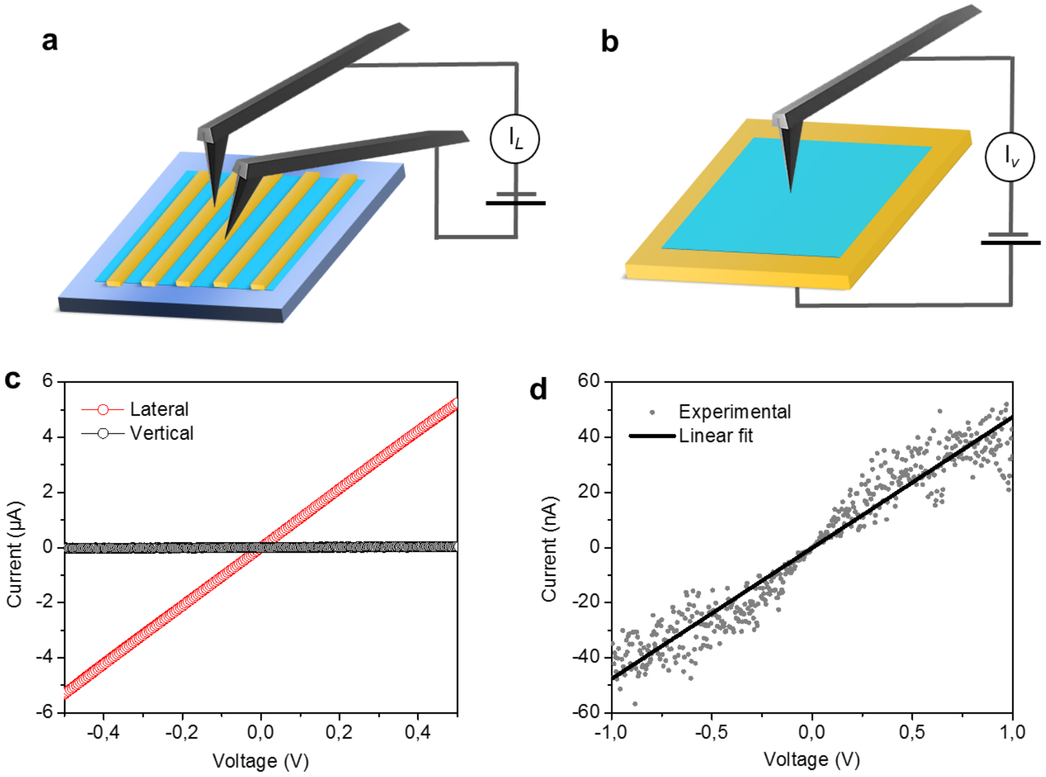


**Supplementary Figure 23 | Electrical conductivity characterization of the q2D PANI film**. (**a-b**) Illustrations of electrical measurements in three directions of electrode construction. The lateral conductivity was measured by two-probe and vertical conductivity by current-sensing AFM. **(c)** *I-V* characteristic curves of the q2D PANI from two probe (lateral) and CS-AFM (vertical). (**d**) The close-up and linear fit of the *I-V* curve of vertical transport. A lateral conductivity $\sigma_L = 0.876$ S $m^{-1}$ and vertical conductivity $\sigma_V = 0.005$ S $m^{-1}$ were obtained respectively. The film is ~9.3 nm thick, and was prepared in 0.2 M HCl aqueous solution using sodium oleyl sulfate monolayer as template.

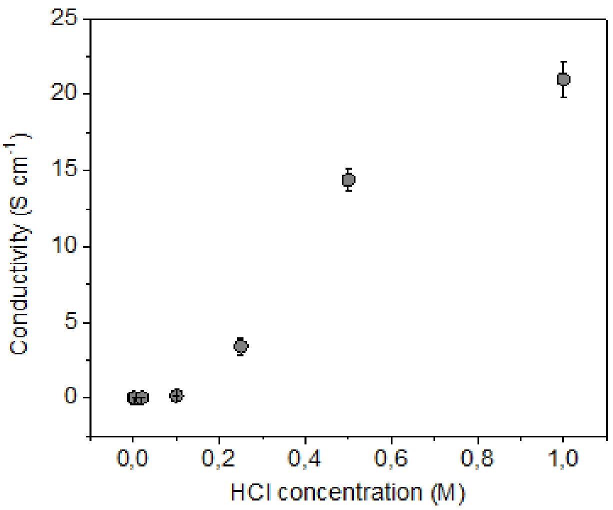


**Supplementary Figure 24 | Lateral conductivity of q2D PANI prepared at different HCl concentrations**.

Error bars indicate the variations in conductivity of each q2D PANI sample at five different positions.

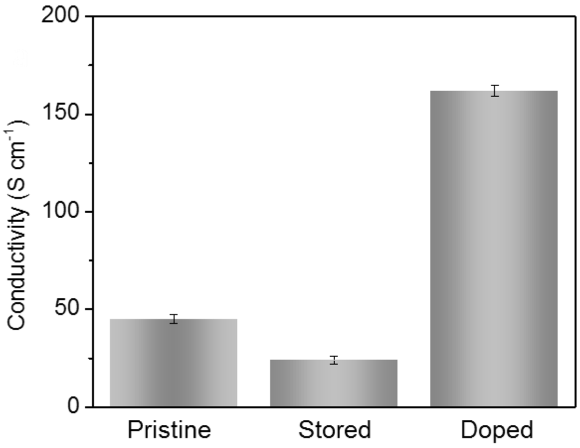


**Supplementary Figure 25 | The stability of electrical conductivity of q2D PANI**. The conductivity of "pristine" q2D PANI was prepared in 1M HCl and measured after drying in vacuum oven for 24 h (at RT). Afterwards, the sample was kept in air for one weeks to measure again the conductivity. The sample was then doped by HCl vapour from concentrated HCl (37%) in a sealed flask for 2 h to achieve a conductivity of ~160 S $cm^{-1}$. The conductivity was measured by Jandel four-point probe system with RM3 test unit. Error bars indicate the variations in conductivity of each q2D PANI sample at five different positions.

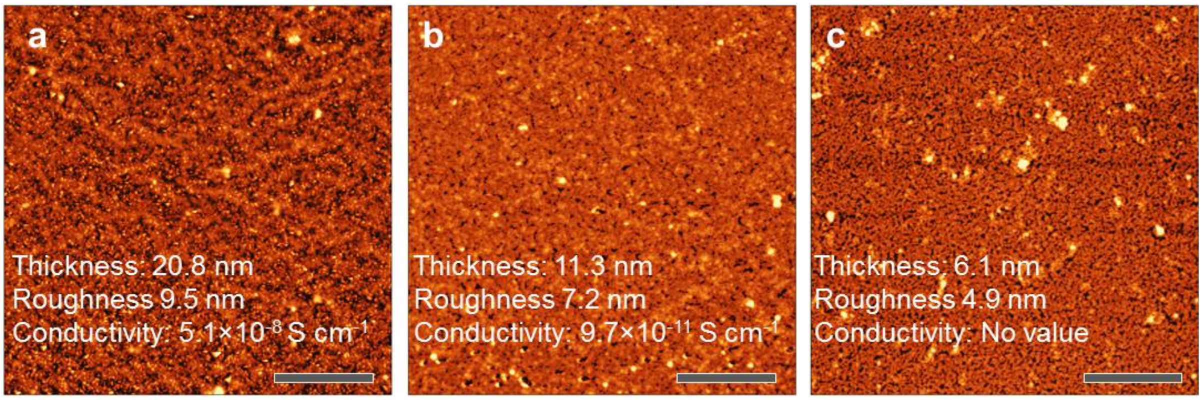


**Supplementary Figure 26 | AFM topographic images of PANI films prepared by spin-coating.** The films of different thickness on 300 nm $SiO_2$/Si were prepared by spin coating from different concentrations of PANI emeraldine base (50000 g $mol^{-1}$) in DMSO: (**a**) 20.8 nm, twice 10 mg $mL^{-1}$; (**b**) 11.3 nm, 10 mg $mL^{-1}$; (**c**) 6.1 nm, 5 mg $mL^{-1}$. Scale bars, 20 µm. The thickness was measured with variable angle spectroscopic ellipsometry at room temperature. The conductivity was measured via the Jandel cylindrical probe combined with the RM3000 test unit. All films were doped in HCl vapour before the conductivity measurement.

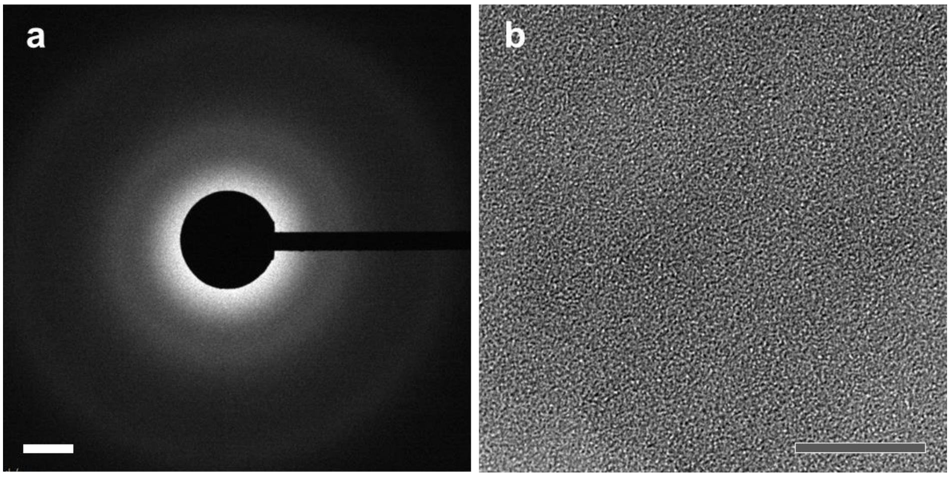


**Supplementary Figure 27 | TEM characterization of PANI film from spin-coating.** (**a**) SAED and (**b**) AC-HRTEM image. Scale bars: (**a**) 2 $nm^{-1}$; (**b**) 10 nm.

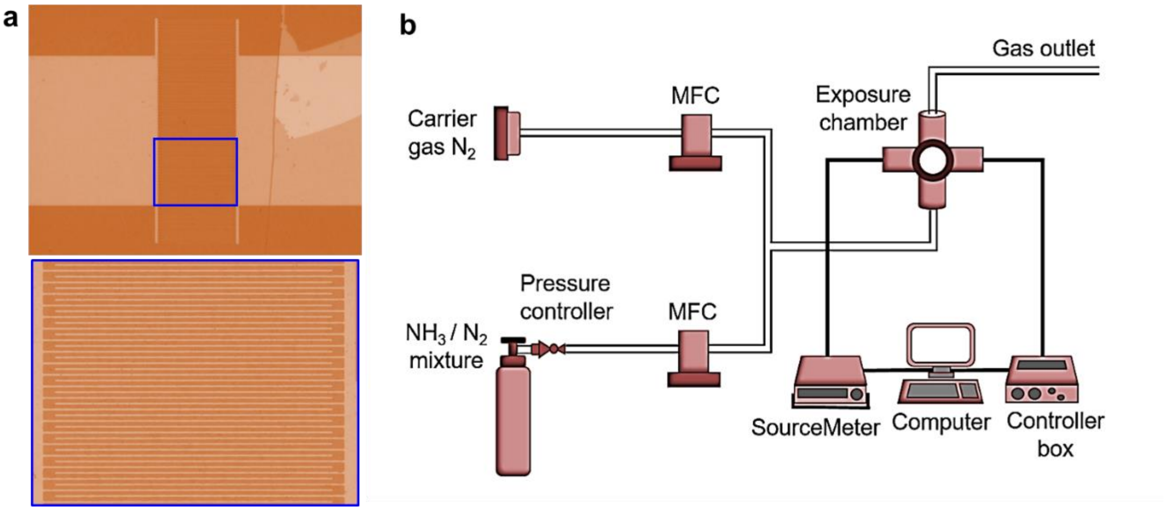


**Supplementary Figure 28 | Demonstration of $NH_3$ sensing testing setup.** (**a**) Optical microscopy image of the sensing area of the $NH_3$ chemiresistor with the q2D PANI on top. (**b**) Sketch of gas detection set up.

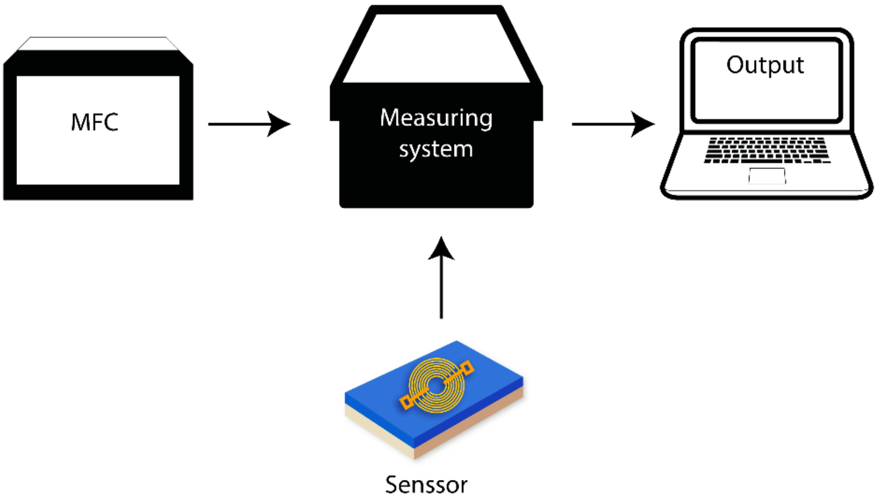


**Supplementary Figure 29 | Sketch of VOCs detection set up.**

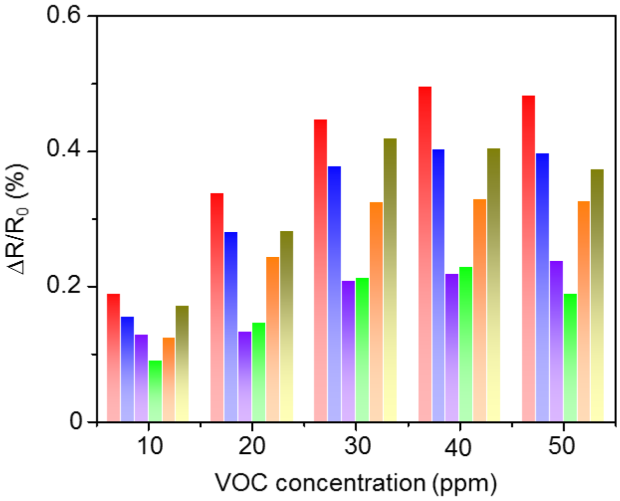


**Supplementary Figure 30 | Column diagram of q2D PANI-based sensors to 3-heptanone.** The q2D PANI sensors were prepared with various dopants and thickness: 0.02 M HCl (~5 nm, red); 0.02 M HCl (~9.3 nm, blue); 0.005 M HCl (~9.3 nm, violet); 0.02 M sulfuric acid (~9.3 nm, green); 0.02 M phytic acid (~9.3 nm, orange); 0.02 M trifluoromethanesulfonic acid (~9.3 nm, yellow).

## Supplementary Tables

**Supplementary Table 1 | Summary of thickness and conductivities of PANI films**. The films were prepared under various amphiphilic monolayers. The reaction time is 48 h and dopant is 0.02 M HCl.

| Surfactants | Film thickness | Conductivity |
|---|---|---|
| 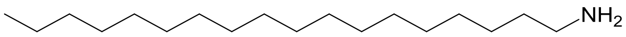 | 6.9 ± 3.5 | No value |
| 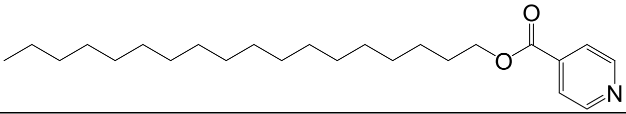 | 8.7 ± 5.3 | No value |
| 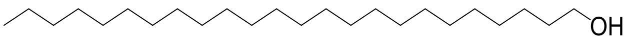 | 11.7 ± 6.8 | $8.30 \pm 0.7 \times 10^{-7}$ S cm$^{-1}$ |
| 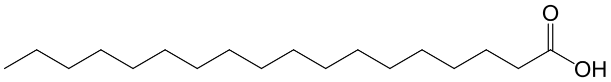 | 8.7 ± 1.8 | $7.25 \pm 0.7 \times 10^{-6}$ S cm$^{-1}$ |
| 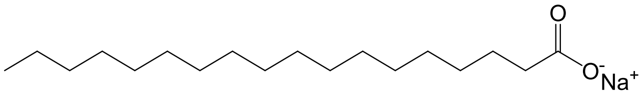 | 9.1 ± 0.9 | $1.20 \pm 0.7 \times 10^{-5}$ S cm$^{-1}$ |
| 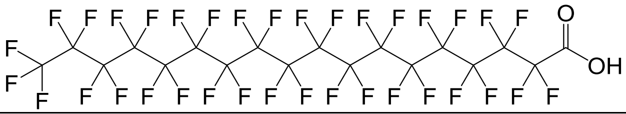 | 9.2 ± 0.8 | $5.03 \pm 0.3 \times 10^{-5}$ S cm$^{-1}$ |
| 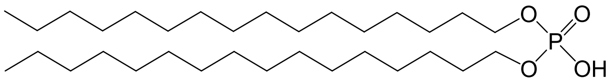 | 10.3 ± 0.5 | $7.82 \pm 0.8 \times 10^{-3}$ S cm$^{-1}$ |
| 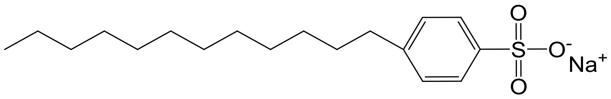 | 10.1 ± 0.4 | $8.59 \pm 1.3 \times 10^{-3}$ S cm$^{-1}$ |
| 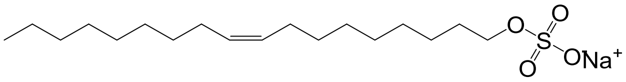 | 9.3 ± 0.3 | $8.76 \pm 0.9 \times 10^{-3}$ S cm$^{-1}$ |
| No surfactant | 13.7 ± 8.4 | No value |

**Supplementary Table 2** | Summary of PANI thin films (< 30 nm) reported in literature.

| **Methods** | **Morphology** | **Lateral size / thickness** | **Crystal structure** | **Lateral conductivity** |
|---|---|---|---|---|
| Ice-templating[1] | 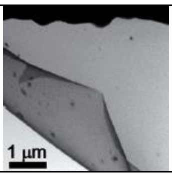 | A few μm / ~30 nm | 60 nm crystal domain | 35 S $cm^{-1}$ |
| SAM-templating[2] | unknown | A few cm / monolayer | unknown | unknown |
| Self-assembly[3] | 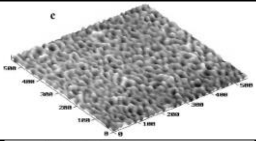 | A few cm / 6 nm | unknown | unknown |
| Self-assembly[4] | 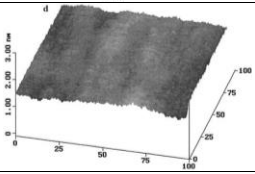 | A few cm / monolayer | unknown | unknown |
| SAM-templating[5] | unknown | A few cm / monolayer | unknown | unknown |
| Langmuir–Blodgett[6] | 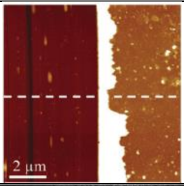 | A few cm / 12.5 nm | unknown | unknown |
| Langmuir–Blodgett[7] | 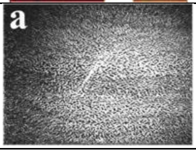 | A few cm / monolayer | unknown | unknown |
| q2D PANI (this work) | 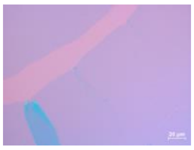 | 50 $cm^2$ / 2.6 - 30 nm | 2.3 μm | 160 S $cm^{-1}$ |

**Supplementary Table 3 |** Literature reported high performance polyaniline based $NH_3$ sensors. The polyaniline-inorganic composites based sensors are excluded.

| **Polyaniline samples** | **$NH_3$ (ppm)** | **Sensitivity (%)** | **Response time (min)** |
|---|---|---|---|
| PANI-HCl nanofibers[8] | 100 | ~2400 | ~25 |
| PANI nanofibers[9] | 100 | ~2900 | ~25 |
| PANI nanotubes[10] | 10 | ~20 | ~33 |
| *t*-Boc PANI nanofibers[11] | 10 | 1.6 | ~16 |
| PANI-DBSA thin film[12] | 5 | ~27 | 2 |
| *In situ* PANI[13] | 5 | ~0.75 | ~1 |
| PANI-AA[14] | 1 | 71 | 2.5 |
| Single PANI nanofiber[15] | 1 | ~8 | ~0.15 |
| PANI-coated MWNTs[16] | 1 | ~5 | 4 |
| PANI/PEO nanowire[17] | 0.8 | ~5 | 0.16 |
| PANI nanoframework[18] | 0.5 | ~60 | ~1 |
| $SiO_2$/PANI nanofibers[19] | 0.4 | 88 | ~9 |
| Thin-film-sc-SWCNT[20] | 0.4 | ~1 | 15 |
| PANI/SWCNTs[21] | 0.05 | ~1.2 | ~20 |
| PANI mesh[22] | 0.0025 | ~2.6 | 2.5 |
| q2D PANI (This work) | 0.03 | 1.85 | 11.2 |

## Supplementary references


1. Choi, I. Y., Lee, J., Ahn, H., Lee, J., Choi, H. C., Park, M. J. High-conductivity two-dimensional polyaniline nanosheets developed on ice surfaces. *Angew. Chem. Int. Edit.* **54**, 10497-10501 (2015).
2. Turyan, I., Mandler, D. Two-dimensional polyaniline thin film electrodeposited on a self-assembled monolayer. *J. Am. Chem. Soc.* **120**, 10733-10742 (1998).
3. Sfez, R., Peor, N., Cohen, S. R., Cohen, H., Yitzchaik, S. In situ SFM study of 2D-polyaniline surface-confined enzymatic polymerization. *J. Mater. Chem.* **16**, 4044-4050 (2006).
4. Sfez, R., De-Zhong, L., Turyan, I., Mandler, D., Yitzchaik, S. Polyaniline monolayer self-assembled on hydroxyl-terminated surfaces. *Langmuir* **17**, 2556-2559 (2001).
5. Kuwabata, S., Fukuzaki, R., Nishizawa, M., Martin, C. R., Yoneyama, H. Electrochemical formation of a polyaniline-analogue monolayer on a gold electrode. *Langmuir* **15**, 6807-6812 (1999).
6. Zhang, J., Burt, D. P., Whitworth, A. L., Mandler, D., Unwin, P. R. Polyaniline Langmuir-Blodgett films: formation and properties. *Phys. Chem. Chem. Phys.* **11**, 3490-3496 (2009).
7. Zhang, J., Mandler, D., Unwin, P. R. Interfacial polymerisation of anilinium at Langmuir monolayers. *Chem. Commun.* **4**, 450-451 (2004).
8. Virji, S., Huang, J. X., Kaner, R. B., Weiller, B. H. Polyaniline nanofiber gas sensors: Examination of response mechanisms. *Nano Lett.* **4**, 491-496 (2004).
9. Huang, J. X., Virji, S., Weiller, B. H., Kaner, R. B. Polyaniline nanofibers: Facile synthesis and chemical sensors. *J. Am. Chem. Soc.* **125**, 314-315 (2003).
10. Gao, Y., Yao, S., Gong, J., Qu, L. Y. Preparation of polyaniline nanotubes via "thin glass tubes template" approach and its gas response. *Macromol. Rapid Commun.* **28**, 286-291 (2007).
11. Uh, K., Kim, T., Lee, C. W., Kim, J. M. A precursor approach to electrospun polyaniline nanofibers for gas sensors. *Macromol. Mater. Eng.* **301**, 1320-1326 (2016).
12. Kumar, J., et al. Highly sensitive chemo-resistive ammonia sensor based on dodecyl benzene sulfonic acid doped polyaniline thin film. *Sci. Adv. Mater.* **7**, 518-525 (2015).
13. Bandgar, D. K., et al. Simple and low-temperature polyaniline-based flexible ammonia sensor: a step towards laboratory synthesis to economical device design. *J. Mater. Chem. C* **3**, 9461-9468 (2015).
14. Chabukswar, V. V., Pethkar, S., Athawale, A. A. Acrylic acid doped polyaniline as an ammonia sensor. *Sens. Actuator B Chem.* **77**, 657-663 (2001).

15. Chen, D. J., Lei, S., Chen, Y. Q. A single polyaniline nanofiber field effect transistor and its gas sensing mechanisms. *Sensors* **11**, 6509-6516 (2011).

16. He, L. F., Jia, Y., Meng, F. L., Li, M. Q., Liu, J. H. Gas sensors for ammonia detection based on polyaniline-coated multi-wall carbon nanotubes. *Mater. Sci. Eng. B-Solid* **163**, 76-81 (2009).

17. Liu, H. Q., Kameoka, J., Czaplewski, D. A., Craighead, H. G. Polymeric nanowire chemical sensor. *Nano Lett.* **4**, 671-675 (2004).

18. Wang, J., et al. Electrochemically fabricated polyaniline nanoframework electrode junctions that function as resistive sensors. *Nano Lett.* **4**, 1693-1697 (2004).

19. Nie, Q. X., et al. Facile fabrication of flexible $SiO_2$/PANI nanofibers for ammonia gas sensing at room temperature. *Colloid Surf. A Physicochem. Eng. Asp.* **537**, 532-539 (2018).

20. Panes-Ruiz, L. A., *et. al.* Toward highly sensitive and energy efficient ammonia gas detection with modified single-walled carbon nanotubes at room temperature. *ACS Sens.* **3**, 79-86 (2018).

21. Zhang, T., Nix, M. B., Yoo, B. Y., Deshusses, M. A., Myung, N. V. Electrochemically functionalized single-walled carbon nanotube gas sensor. *Electroanalysis* **18**, 1153-1158 (2006).

22. Cai, J. X., Zhang, C. P., Khan, A., Liang, C. W., Li, W. D. Highly transparent and flexible polyaniline mesh sensor for chemiresistive sensing of ammonia gas. *RSC Adv.* **8**, 5312-5320 (2018).
